# Prevalence and correlates of undiagnosed, diagnosed, and total type 2 diabetes among adults in Morocco, 2017

**DOI:** 10.1038/s41598-022-20368-4

**Published:** 2022-09-27

**Authors:** Supa Pengpid, Karl Peltzer

**Affiliations:** 1 Department of Health Education and Behavioral Sciences, Faculty of Public, Health Mahidol University, Bangkok, Thailand; 2 Department of Public Health, Sefako Makgatho Health Sciences University, Pretoria, South Africa; 3 Department of Healthcare Administration, College of Medical and Health Science, Asia University, Taichung, Taiwan; 4 Department of Psychology, College of Medical and Health Science, Asia University, Taichung, Taiwan

**Keywords:** Diseases, Health care

## Abstract

The study aimed to estimate the prevalence and associated factors of undiagnosed type 2 diabetes (T2D) among adults in Morocco. Cross-sectional data were analyzed from 4779 people (≥ 18 years, mean age 41.7 years) who participated in the Morocco STEPS nationally representative survey in 2017 and had completed fasting blood glucose measurement. The results indicate that the prevalence of undiagnosed T2D was 5.9% (44.7% of total T2D), diagnosed T2D 7.3% and total T2D 13.2%. In the adjusted multinomial logistic regression analysis, older age (≥ 50 years), receipt of health care advice, and obesity were positively associated with undiagnosed T2D. Older age (≥ 50 years), urban residence, receipt of health care advice, ever cholesterol screening, moderate sedentary behaviour, obesity, hypertension, and elevated total cholesterol were positively associated with diagnosed T2D. In adjusted logistic regression analysis, older age (≥ 50 years), receipt of health care advice and cholesterol screening were negatively associated with undiagnosed T2D versus diagnosed T2D. A significant proportion of adults in Morocco had undiagnosed T2D and several associated factors were identified that can help guide interventions.

## Introduction

According to the World Health Organization^1^, more than one and a half million people died from diabetes in 2019; however, diabetes can be treated with diet, physical activity, medication and regular screening and management of complications. Untreated undiagnosed type 2 diabetes (T2D) may have serious consequences, including microvascular and macrovascular complications^2,3^ and an increased risk of mortality^4,5^, highlighting the crucial importance of early diagnosis. In 2021, worldwide 44.7% of adults with T2D did not know they had T2D^6^. In an earlier review of studies in 29 low- and middle-income countries (LMICs), the prevalence of undiagnosed T2D was 4.9%^7^, and in a review of 55 studies in the eastern Mediterranean region, the pooled prevalence of undiagnosed T2D was 5.45%, for example, in Tunisia 7.7% and in Iraq 6.2%^8^. In a small sample of urban Sahraoui women in South Morocco, the prevalence of undiagnosed T2D was 6.4% in 2001/2002^9^. Due to 44% of the diabetes population being undiagnosed in Morocco^10^, the Moroccan Ministry of Health launched a public awareness campaign in 2015 on the different types of diabetes, its risk factors, warning signs and complications, the importance of screening and the adoption of a healthy lifestyle, involving health professionals and other lay persons in awareness and health education and involving the media in information and awareness on diabetes and its complications^11^. There is a lack of national data on undiagnosed T2D and its correlates in Morocco^8,12^. These data may help to assess public health interventions related to the screening and diagnosis of T2D in Morocco.

Predisposing factors associated with undiagnosed T2D included in some studies younger adults^13–15^, older adults^16–19^, male sex^15,17^, living alone ^15,20^, and family history of diabetes^21,22^. Enabling/disabling factors associated with undiagnosed T2D include rural residence^13,16^, in some studies lower economic status^13,23^, food insecurity^24^, and lower education^14,25^, and in other studies higher economic status^17^, and higher education^23^. Other enabling/disabling factors associated with undiagnosed T2D include knowing symptoms of diabetes^21^, no health care visit in the past 12 months^15^, health insurance status (having medical insurance^16^, and not having private insurance^26^ and health risk behaviours (high sedentary behaviour^27^, heavy alcohol use^28^, and high level of physical activity^23^.

The need factors associated with undiagnosed T2D include other chronic diseases^16^, hypertension status (not hypertension^13,14^, hypertension^17,18,29,30^), obesity^17–19,21,22,28,31^, low HDL-C^31,32^, high triglycerides^31,32^, dyslipidaemia^22^, cardiovascular disease status (not heart disease^14^, cardiovascular disease^18^), and perceived poor health^16^. The study aimed to estimate the prevalence and associated factors of undiagnosed T2D among people 18 years and older in Morocco.

## Methods

### Study design and participants

Secondary data from the ‘STEPwise approach to NCD risk factor Surveillance’ (STEPS) cross-sectional survey in Morocco in 2017^33^ with complete measurements of fasting blood glucose were analyzed; the overall response rate was 89.0%^34^. STEPS focus is on “obtaining population-based data on the established risk factors that determine the major disease burden on a regular basis”^33^. Participants were randomly selected from the target population (18 years and older), using a multi-stage stratified sampling procedures^34^. Following the STEP-wise survey procedures, in Step 1 behavioural and sociodemographic data were collected. In Step 2, physical measurements and blood pressure were assessed, and in Step 3. biochemical measurements were collected to assess blood glucose and cholesterol^33^. Blood glucose, total cholesterol and triglycerides were measured in peripheral (capillary) blood at the data collection site; equipment used was the Cardiochek® PA (pts Diagnostics, Indianapolis, Indiana, USA) with a Chip MeMo, Blood Glucose Strips and lipids^34^.

Ethics approval was provided by the Biomedical Research Ethics Committee, Faculty of Medicine and Pharmacy of Rabat, Morocco, and participants provided written informed consent. All methods were performed in accordance with the relevant guidelines and regulations.

## Measures

### Outcome variable

Undiagnosed T2D was defined as fasting plasma glucose level ≥ 126 mg/dL among people who responded “no” to the question “Have you ever been told by a doctor or other health worker that you have raised blood sugar or diabetes?” Diagnosed T2D was defined as those who answered “yes” to the question of ever having been told by a health care worker that they had diabetes, and total T2D included those with undiagnosed and diagnosed T2D^35^.

*Predisposing factors* included age, sex, and marital status.

*Enabling or disabling factors* included residence status, receipt of health care advice, ever screening for cholesterol, smoking tobacco history, physical activity, and sedentary behaviour. The receipt of health care advice was assessed with the question, “During the past three years, has a doctor or other health worker advised you to maintain a healthy body weight or lose weight?” (yes/no). Smoking history was assessed with two questions, “Do you currently smoke any tobacco products, such as cigarettes, cigars or pipes?” (Yes, No) and “In the past, did you ever smoke any tobacco products?”(Yes, No)^34^. Self-reported physical activity and sedentary behaviour were assessed with the Global Physical Activity Questionnaire (GPAQ) and categorized by the median metabolic equivalent (METs) of performed activities as low, moderate, and high^36^, and sedentary behaviour defined as low (< 4 h), moderate (4 to < 8 h) and high (≥ 8 h sitting/day)^37^.

*The need factors* included BMI, hypertension, heart attack or stroke, and elevated total cholesterol. Body mass index (BMI) was classified as “underweight (< 18.5 kg/m^2^), normal weight (18.5–24.9 kg/m^2^), overweight (25.0–29.9 kg/m^2^), and obesity (≥ 30.0 kg/m^2^)”^33^. Hypertension was defined as “systolic BP ≥ 140 mmHg and/or diastolic BP ≥ 90 mmHg and/or previous or current treatment with antihypertensive drugs”^38^. History of heart attack or stroke included self-reported “Have you ever had a heart attack or chest pain from heart disease (angina) or a stroke (cerebrovascular accident or incident)? (Yes, No)”^34^. Elevated total cholesterol was classified^39^ as: “being on antilipidemic medication or having elevated total cholesterol (TC): ≥ 5.17 mmol/l (200 mg/dl)”.

## Sample size calculations and data analysis

Sample size calculation. This data set had an overall N = 5429 and 4779 (88%) persons from the total sample had completed fasting blood glucose measurement. From a review in the study region, the pooled prevalence of undiagnosed T2D was 5.45%^8^. We calculated the sample size with Epi-Info with population 100,000, expected frequency 5.45, acceptable margin of error 5% confidence level 99.99, minimum sample size is 317. In our study we calculated that based on the existing data set of 4799 people, the sample size is sufficient.

All statistical analyses were conducted with STATA software version 14.0 (Stata Corporation, College Station, TX, USA). Analysis weights were calculated by taking the inverse of the probability of selection of each participant adjusted for differences in the age-sex composition of the sample population compared to the target population.^33^ Descriptive statistics are used to describe the sample. Pearson chi-square statistics were used to calculate differences in proportion. Multinomial logistic regression was used to estimate factors associated with undiagnosed T2D and diagnosed T2D (with not having T2D as reference category). Logistic regressions were used to assess the associations with undiagnosed T2D versus diagnosed T2D. Covariates in the logistic regression models included predisposing factors (age, gender, and marital status), enabling, or disabling factors (residence status, reported receipt of health care advice, cholesterol screening, education, smoking status, physical activity, and sedentary behaviour) and need factors (BMI, hypertension, heart attack or stroke, and elevated total cholesterol). Variables significant in univariable analyses were subsequently included in the multivariable models. To account for the multi-stage sample design, Taylor linearization methods were utilized. P-values < 0.05 were considered significant and missing values were discarded.

## Results

### Sample characteristics

The sample with complete fasting blood glucose measurement included 4779 persons (≥ 18 years), with a mean age of 41.7 years (SD = 16.5 years) in 2017. The prevalence of undiagnosed T2D was 5.9% (44.7% of total T2D), diagnosed T2D 7.3%, and total T2D 13.2%. Further sociodemographic and health characteristics of the sample by T2D status are described in Table 1.

**Table 1:** Characteristics and predisposing, enabling/disabling and risk factors in a sample of 4779 adults screened for diagnosed and undiagnosed diabetes in Morocco, 2017.

Variable	Sample	No diabetes	Undiagnosed T2D	Diagnosed T2D	*p*-value^a^
N	4779	4003	333	443	
	N (%)	%	%	%	
All		86.8	5.9	7.3	
**Predisposing factors**					
Age (years)					
18–34	1343 (38.9)	95.1	3.4	1.6	< 0.001
35–49	1546 (30.4)	89.3	5.5	5.1	
50 or more	1890 (30.7)	73.8	9.7	16.6	
Gender					
Female	3139 (50.9)	84.4	7.1	8.5	< 0.001
Male	1640 (49.1)	89.2	4.8	6.0	
Marital status					
Not married	1250 (31.1)	87.9	5.8	6.3	0.266
Married	3525 (68.9)	86.4	6.0	7.7	
**Enabling/disabling factors**					
Residence					
Rural	1909 (36.3)	90.5	5.3	4.2	< 0.001
Urban	2870 (63.7)	84.7	6.3	9.0	
Receipt of health care advice					
No	3329 (71.3)	91.4	4.8	3.7	< 0.001
Yes	1450 (28.7)	75.3	8.7	16.0	
Ever cholesterol screening					
No	4099 (88.5)	89.4	5.8	4.8	< 0.001
Yes	680 (11.5)	66.7	6.9	26.3	
Education					
None	2468 (41.1)	82.3	7.7	10.0	< 0.001
Primary	1003 (23.1)	88.5	5.4	6.1	
> Primary	1305 (35.9)	90.9	4.3	4.8	
Smoking tobacco					
Never	4076 (80.6)	86.0	6.4	7.6	0.002
Past	346 (8.5)	87.1	4.0	8.9	
Current	357 (10.9)	92.3	4.4	3.3	
Physical activity					
Low	1249 (24.8)	82.8	7.3	9.9	< 0.001
Moderate	1037 (21.8)	83.5	7.0	9.5	
High	2476 (53.4)	90.0	4.9	5.1	
Sedentary behaviour					< 0.001
Low	3180 (66.3)	88.3	5.8	5.9	
Moderate	1291 (27.6)	84.4	6.3	9.3	
High	299 (6.0)	81.5	5.6	5.6	
**Need factors**					
Body mass index					
Underweight	187 (5.6)	95.8	3.1	1.1	< 0.001
Normal	1681 (40.6)	91.2	4.7	4.1	
Overweight	1635 (33.3)	85.6	6.0	8.5	
Obesity	1142 (20.4)	76.7	9.7	13.6	
Hypertension	1083 (19.9)	76.0	8.4	15.6	< 0.001
Heart attack or stroke	169 (3.3)	78.5	5.7	15.8	< 0.001
Elevated total cholesterol	421 (7.4)	76.4	8.7	14.9	< 0.001

^a^Based on chi-square statistics.

### Associations with undiagnosed and diagnosed T2D versus non-diabetic

In the adjusted multinomial logistic regression analysis, older age (≥ 50 years) (ARRR: 2.80, 95% CI: 1.80–4.35), receipt of health care advice (ARRR: 1.79, 95% CI: 1.32–2.41), and obesity (ARRR: 1.71, 95% CI: 1.19–2.46) were positively associated with undiagnosed T2D. Older age (≥ 50 years) (ARRR: 6.64, 95% CI: 3.81–11.60), urban residence (ARRR: 1.50, 95% CI: 1.12–2.00), receipt of health care advice (ARRR: 2.92, 95% CI: 2.23–3.82), ever cholesterol screening (ARRR: 2.64, 95% CI: 1.99–3.50), moderate sedentary behaviour (ARRR: 1.33, 95% CI: 1.01–1.75), obesity (ARRR: 1.56, 95% CI: 1.11–2.19), hypertension (ARRR: 1.47, 95% CI: 1.13–1.92), and elevated total cholesterol (ARRR: 2.64, 95% CI: 1.99–3.50) were positively associated with diagnosed T2D. In addition, in unadjusted analyses, male sex, higher education, and high physical activity were negatively associated, and hypertension and elevated total cholesterol were positively associated with undiagnosed T2D (see Tables 2 and 3).

**Table 2:** Relative risk ratios for factors associated with undiagnosed and diagnosed type 2 diabetes (T2D) in Moroccan adults, 2017.

Variable	Undiagnosed T2D	Diagnosed T2D
	Unadjusted RRR (95% CI)	Unadjusted RRR (95% CI)
**Predisposing factors**		
Age (years)		
18–34	1 (Reference)	1 (Reference)
35–49	1.75 (1.15–2.66)**	3.70 (2.54–5.40)***
50 or more	3.70 (2.54–5.40)***	13. 74 (8.28–22.79)***
Gender		
Female	1 (Reference)	1 (Reference)
Male	0.65 (0.49–0.85)**	0.66 (0.53–0.84)***
Marital status		
Not married	1 (Reference)	1 (Reference)
Married	1.04 (0.78–1.39)	1.24 (0.97–1.60)
**Enabling/disabling factors**		
Residence		
Rural	1 (Reference)	1 (Reference)
Urban	1.27 (0.97–1.66)	2.32 (1.81–2.98)***
Receipt of health care advice		
No	1 (Reference)	1 (Reference)
Yes	2.21 (1.69–2.87)***	5.21 (4.16–6.53)***
Ever cholesterol screening		
No	1 (Reference)	1 (Reference)
Yes	1.61 (1.12–2.31)**	7.39 (5.86–9.33)***
Education		
None	1 (Reference)	1 (Reference)
Primary	0.65 (0.46–0.91)*	0.57 (0.42–0.76)***
> Primary	0.50 (0.36–0.70)***	0.44 (0.33–0.58)***
Smoking tobacco		
Never	1 (Reference)	1 (Reference)
Past	0.62 (0.36–1.06)	1.14 (0.79–1.66)
Current	0.64 (0.37–1.14)	0.39 (0.23–0.65)***
Physical activity		
Low	1 (Reference)	1 (Reference)
Moderate	0.94 (0.67–1.32)	0.94 (0.71–1.25)
High	0.61 (0.45–0.83)**	0.47 (0.37–0.62)***
Sedentary behaviour		
Low	1 (Reference)	1 (Reference)
Moderate	1.13 (0.85–1.50)	1.64 (1.29–2.09)***
High	1.05 (0.63–1.75)	2.34 (1.61–3.40)***
**Need factors**		
Body mass index		
< 25 kg/m^2^	1 (Reference)	1 (Reference)
Overweight	1.45 (1.04–2.01)*	2.39 (1.78–3.21)***
Obesity	2.62 (1.89–3.61)***	4.32 (3.23–5.77)***
Hypertension	2.82 (1.40–2.40)***	3.55 (2.84–4.44)***
Heart attack or stroke	1.05 (0.51–2.14)	2.51 (1.60–3.92)***
Elevated total cholesterol	1.77 (1.19–2.63)**	2.50 (1.84–3.40)***

*RRR* Relative, *CI* Confidence intervals.

*p < 0.05; **p < 0.01; ***p < 0.001.

**Table 3:** Relative risk ratios for factors associated with undiagnosed and diagnosed type 2 diabetes (T2D) in Moroccan adults, 2017, adjusted for age, gender,

Variable	Undiagnosed T2D	Diagnosed T2D
	Adjusted RRR (95% CI)	Adjusted RRR (95% CI)
**Predisposing factors**		
Age (years)		
18–34	1 (Reference)	1 (Reference)
35–49	1.44 (0.92–2,24)	2.58 (1.46–4.57)***
50 or more	2.80 (1.80–4.35)***	6.64 (3.81–11.60)***
Gender		
Female	1 (Reference)	1 (Reference)
Male	0.84 (0.59–1.21)	1.02 (0.73–1.43)
**Enabling/disabling factors**		
Residence		
Rural	1 (Reference)	1 (Reference)
Urban	1.13 (0.83–1.54)	1.50 (1.12–2.00)**
Receipt of health care advice		
No	1 (Reference)	1 (Reference)
Yes	1.79 (1.32–2.41)***	2.92 (2.23–3.82)***
Ever cholesterol screening		
No	1 (Reference)	1 (Reference)
Yes	0.84 (0.55–1.27)	2.64 (1.99–3.50)***
Education		
None	1 (Reference)	1 (Reference)
Primary	0.94 (0.65–1.37)	0.95 (0.67–1.35)
> Primary	0.75 (0.51–1.09)	0.75 (0.53–1.05)
Smoking tobacco		
Never	1 (Reference)	1 (Reference)
Past	0.59 (0.32–1.09)	0.80 (0.49–1.29)
Current	0.87 (0.45–1.68)	0.64 (0.35–1.18)
Physical activity		
Low	1 (Reference)	1 (Reference)
Moderate	1.09 (0.77–1.56)	1.14 (0.83–1.56)
High	0.82 (0.60–1.13)	0.94 (0.69–1.26)
Sedentary behaviour		
Low	1 (Reference)	1 (Reference)
Moderate	1.03 (0.77–1.38)	1.33 (1.01–1.75)*
High	0.82 (0.48–1.40)	1.53 (0.99–2.37)
**Need factors**		
Body mass index		
< 25 kg/m^2^	1 (Reference)	1 (Reference)
Overweight	1.14 (0.81–1.61)	1.29 (0.93–1.80)
Obesity	1.71 (1.19–2.46)**	1.56 (1.11–2.19)**
Hypertension	1.21 (0.89–1.64)	1.47 (1.13–1.92)**
Heart attack or stroke	0.92 (0.45–1.91)	1.57 (0.92–2.69)
Elevated total cholesterol	1.39 (0.89–2.15)	2.64 (1.99–3.50)***

*RRR*  Relative risk ratio, *CI* Confidence intervals.

*p < 0.05; **p < 0.01; ***p < 0.001.

### Associations with undiagnosed T2D versus diagnosed T2D

In adjusted logistic regression analysis, older age (≥ 50 years) (AOR: 0.32, 95% CI: 0.16–0.64), receipt of health care advice (AOR: 0.51, 95% CI: 0.36–0.72), and ever cholesterol screening (AOR: 0.31, 95% CI: 0.20–0.47) were negatively associated with undiagnosed T2D versus diagnosed T2D. Furthermore, in the unadjusted analysis, urban residence, sedentary behaviour, obesity, hypertension, and heart attack or stroke were negatively associated with undiagnosed T2D versus diagnosed T2D (see Table 4).

**Table 4:** Unadjusted and adjusted Odds Ratios (OR)for factors associated with diagnosed or undiagnosed type 2 diabetes (T2D) in Moroccan adults, 2017.

Variable	Unadjusted OR (95% CI)	Adjusted OR (95% CI)
**Predisposing factors**		
Age (years)		
18–34	1 (Reference)	1 (Reference)
35–49	0.50 (0.25–0.98)*	0.43 (0.20–0.92)*
50 or more	0.27 (0.15–0.50)***	0.32 (0.16–0.64)***
Gender		
Female	1 (Reference)	–
Male	0.97 (0.69–1.37)	
Marital status		
Not married	1 (Reference)	–
Married	0.84 (0.58–1.21)	
**Enabling/disabling factors**		
Residence		
Rural	1 (Reference)	1 (Reference)
Urban	0.55 (0.38–0.78)***	0.81 (0.55–1.19)
Receipt of health care advice		
No	1 (Reference)	1 (Reference)
Yes	0.42 (0.31–0.59)***	0.51 (0.36–0.72)***
Ever cholesterol screening		
No	1 (Reference)	1 (Reference)
Yes	0.22 (0.15–0.33)***	0.31 (0.20–0.47)***
Education		
None	1 (Reference)	–
Primary	1.15 (0.75–1.76)	
> Primary	1.15 (0.77–1.73)	
Smoking tobacco		
Never	1 (Reference)	–
Past	0.54 (0.29–1.01)	
Current	1.66 (0.79–3.48)	
Physical activity		
Low	1 (Reference)	–
Moderate	1.00 (0.66–1.50)	
High	1.29 (0.88–1.89)	
Sedentary behaviour		
Low	1 (Reference)	1 (Reference)
Moderate	0.69 (0.48–0.98)*	0.74 (0.50–1.11)
High	0.45 (0.25–0.81)**	0.52 (0.27–1.01)
**Need factors**		
Body mass index		
< 25 kg/m^2^	1 (Reference)	1 (Reference)
Overweight	0.60 (0.39–0.92)*	0.83 (0.52–1.32)
Obesity	0.61 (0.40–0.91)*	1.07 (0.67–1.70)
Hypertension	0.51 (0.37–0.71)***	0.80 (0.56–1.14)
Heart attack or stroke	0.42 (0.19–0.93)*	0.61 (0.23–1.61)
Elevated total cholesterol	0.71 (0.44–1.12)	–

*OR* Odds ratio, *CI* Confidence intervals.

*p < 0.05; **p < 0.01; ***p < 0.001.

## Discussion

The study found a national prevalence of undiagnosed T2D (5.9%, 44.7% of total T2D), which is similar to recent global Figs. (44.7% of total T2D)^6^, higher than in an earlier review of studies in 29 LMICs (4.9%)^7^, higher than in a review of studies in the Eastern Mediterranean region (5.45%)^8^, and lower than in a local study among women in Morocco (6.4%)^9^. People with undiagnosed T2D versus diagnosed T2D showed fewer diabetes-related risk factors, such as younger age, no obesity, and no hypertension than those with diagnosed T2D. This may be explained by people with undiagnosed T2D being generally younger and healthier than those with diagnosed T2D, mostly at an earlier stage of T2D^40^.

Consistent with some previous research^14–19^, some predisposing factors (older age) were associated with undiagnosed T2D versus no T2D. According to some previous studies^15,16,26^, enabling / disabling factors associated with undiagnosed T2D versus diagnosed T2D included no receipt of health care advice (to lose weight) in the past three years, and had never been screened for cholesterol. People with receipt of health care advice and cholesterol screening are more likely to use health services and may consequently reduce the odds of undiagnosed T2D^16^. Following the T2D management guidelines in Morocco, patients with diabetes are expected to attend health care services more often^41^, which may explain that people with diagnosed T2D visit health care providers more often than people with undiagnosed T2D^15^. While some studies^13,16^ found an association between rural residence and undiagnosed T2D, we only found this association in unadjusted analysis. Previous studies^14,23,25^ found mixed results regarding the association between educational status and undiagnosed T2D, while we found no significant association.

Consistent with previous research^17–19,21,22,28^, need factors associated with undiagnosed T2D included other chronic diseases, such as obesity. Obesity is a known risk factor for diabetes^21^. Consistent with some research^13–15^, in unadjusted analysis, we found a negative association between hypertension, cardiovascular disease, and undiagnosed T2D versus diagnosed T2D. Unlike some previous studies^22,31,32^, we did not find an association between elevated total cholesterol and undiagnosed T2D. However, we found a statistically significant positive association between elevated total cholesterol and diagnosed T2D. This may be explained by people with diagnosed T2D who are generally older and less healthy, mostly at a later stage of T2D than those with undiagnosed T2D^40^.

### Strengths and limitations

The study strengths include the use of nationally representative adult sample of all ages and standardized STEPS methodology and measures. The limitation of the study is using peripheral (capillary) blood instead venous plasma glucose^42^. However, Priya et al.^43^ showed that capillary blood glucose is a feasible alternative for screening for diabetes in epidemiological studies in developing countries where obtaining venous samples may be difficult. Some variables were evaluated by self-report, which may have biased responses, and the cross-sectional design precludes causative conclusions between the evaluated variables. The sample only included those persons who were non-institutionalized, while the inclusion of institutionalized persons would have given different estimates. Furthermore, certain variables, such as knowledge of diabetes symptoms and a family history of diabetes, were not evaluated and should be included in future research.

### Implications for public health research and practice

Policy implications are that increased public awareness campaigns, and screening of T2D are needed to reduce undiagnosed T2D in Morocco. The Morocco national NCD programme includes community awareness campaigns on diabetes, screening/early detection, and integrated care for diabetes^44^.

## Conclusion

A significant proportion of adults in Morocco had undiagnosed T2D. Predisposing factors (older age), enabling factors (receipt of health care advice) and need factors (obesity) were identified as associated with undiagnosed T2D versus no T2D, and predisposing factors (younger age), and enabling / enabling factors (no receipt of health care advice, never been screened for cholesterol) were identified as associated with undiagnosed T2D versus diagnosed T2D, which can be targeted in interventions.

## Data Availability

The data source is publicly available at the World Health Organization NCD Microdata Repository (URL: https://extranet.who.int/ncdsmicrodata/index.php/catalog).
